# The response of small boreal catchments to extreme weather event: Hurricane Larry

**DOI:** 10.1371/journal.pone.0345113

**Published:** 2026-04-13

**Authors:** Kavi M. Heerah, Kailee G. Clarke, Heather E. Reader

**Affiliations:** 1 Environmental Sciences Program, Memorial University of Newfoundland and Labrador, St. John’s, Canada; 2 Department of Chemistry, Memorial University of Newfoundland and Labrador, St. John’s, Canada; National Research and Innovation Agency, INDONESIA

## Abstract

The boreal environment is high in dissolved organic carbon (DOC) and iron concentrations. This DOC is enriched in functional groups allowing it to bind strongly with iron and act as a significant source of iron to the coastal and marine environment. As climate change intensifies more extreme weather events will affect the northern hemisphere and boreal environment. These weather events can lead to massive fluxes of material but the impact it will have is currently unknown. Hurricane Larry made landfall on Newfoundland (NL) in 2021 providing an opportunity to investigate how the boreal environment will react to extreme weather events. We sampled three rivers before and after the hurricane to see how DOC and iron concentrations, and colour (a_350_) were affected by the hurricane. Temperature and pH were found to decrease in a statistically significant manner following the storm with iron and a_350_ having a significant increase, DOC despite increasing following the storm failed to meet the statistical significance threshold. The rivers chosen had a high abundance of natural landcover which buffered the catchment’s response from the hurricane. Pearson’s correlations were calculated between the landcover and the ∆ of the variables to access the resilience of the catchments to extreme weather events. Due to the small sample size Pearson’s corelations could not achieve statistical significance but the results still provide a novel look into the resilience of the northern boreal regions to extreme weather events. These preliminary results show that a high percentage of forest and peatlands buffered against increases in DOC and colour, with wetlands buffering an increase in iron concentrations. This study represents one of the first to observe boreal catchment responses to extreme weather events such as hurricanes and can serve as basis for more robust studies based in the boreal region.

## 1. Introduction

Dissolved organic matter (DOM) is a complex mixture of compounds of diverse biological origins and diagenetic states. Rivers deliver significant amounts of terrestrial DOM and other important macro- and micro- nutrients to the coastal ocean, globally [1–3]. Terrestrial DOM impacts the coastal ecosystem by acting as a substrate for microbial degradation processes, and limiting light penetration, tipping the balance towards net heterotrophy [4–7]. Terrestrial DOM brings with it a number of important nutrients, and in particular is recognized as an important supply of dissolved iron (dFe), thereby increasing coastal productivity [3,8].

High intensity storm events such as hurricanes have been shown to cause increases in terrestrial DOM loading in rivers and estuaries [9–12]. A number of studies have found that significant amounts of a river’s annual DOM load can be exported as a result of a major storm event, in some cases up to 50% or more [12–16]. These massive fluxes of DOM occur over short time periods, and can have drastic consequences for coastal carbon cycling [10,17–19]. Yan et al. (2020) found that the DOM flux associated with Hurricane Harvey in 2017 increased carbon cycling in Galveston Bay, Texas (USA) with the majority of the flux mineralized within a month of the storm. On the other hand, Avery et al. (2004) found that the majority of the riverine DOM associated with Hurricane Floyd in 1999 (Long Bay, North Carolina, USA) increased the concentration of recalcitrant DOM in Long Bay. Regardless of the fraction of bioavailable DOM for each event, the total fluxes of DOM and freshwater are such that the coastal bacterial community is altered [9,17,20]. The impact of these storm events persist in the coastal environment for long periods of time from days to months [9,10,13]. As climate change and the warming of the ocean continues high intensity weather events, such as hurricanes are likely to affect more northern regions, such as the boreal environment [21–25]. The majority of studies investigating hurricanes and extreme weather events are based in more southern regions. The response of the boreal environment to hurricanes has not been studied before given its previously rare occurrence.

The boreal environment is a rich source of carbon and iron to the coastal/ marine environment. Changes in the export of material from the boreal environment can have impacts on terrestrial biogeochemical cycles and the nearby coastal communities. DOM from boreal and peatland ecosystems have been identified as important for biogeochemical nutrient cycling [3,8,26]. The acidic soils in boreal forest and the anoxic conditions present in peat barrens can allow for the accumulation of iron in the soils [27,28]. Peat and boreal forest produce DOM enriched in aromatics, lignins, humic acids, and humic ligands [26,29]. This DOM can form complexes with iron more resistant to flocculation allowing greater terrestrial inputs of iron and carbon into the coastal environment [3,30–32]. DOM concentrations have been increasing in inland waters across the boreal region due at least in part to warming conditions and increased precipitation, leading to brownification of waterways and increasing terrestrial inputs of DOM to the coastal ocean [33–38]. The impact of the convergence of brownification and extreme weather events is currently unknown but clearly has the potential to impact the coastal environment.

**Fig 1:**
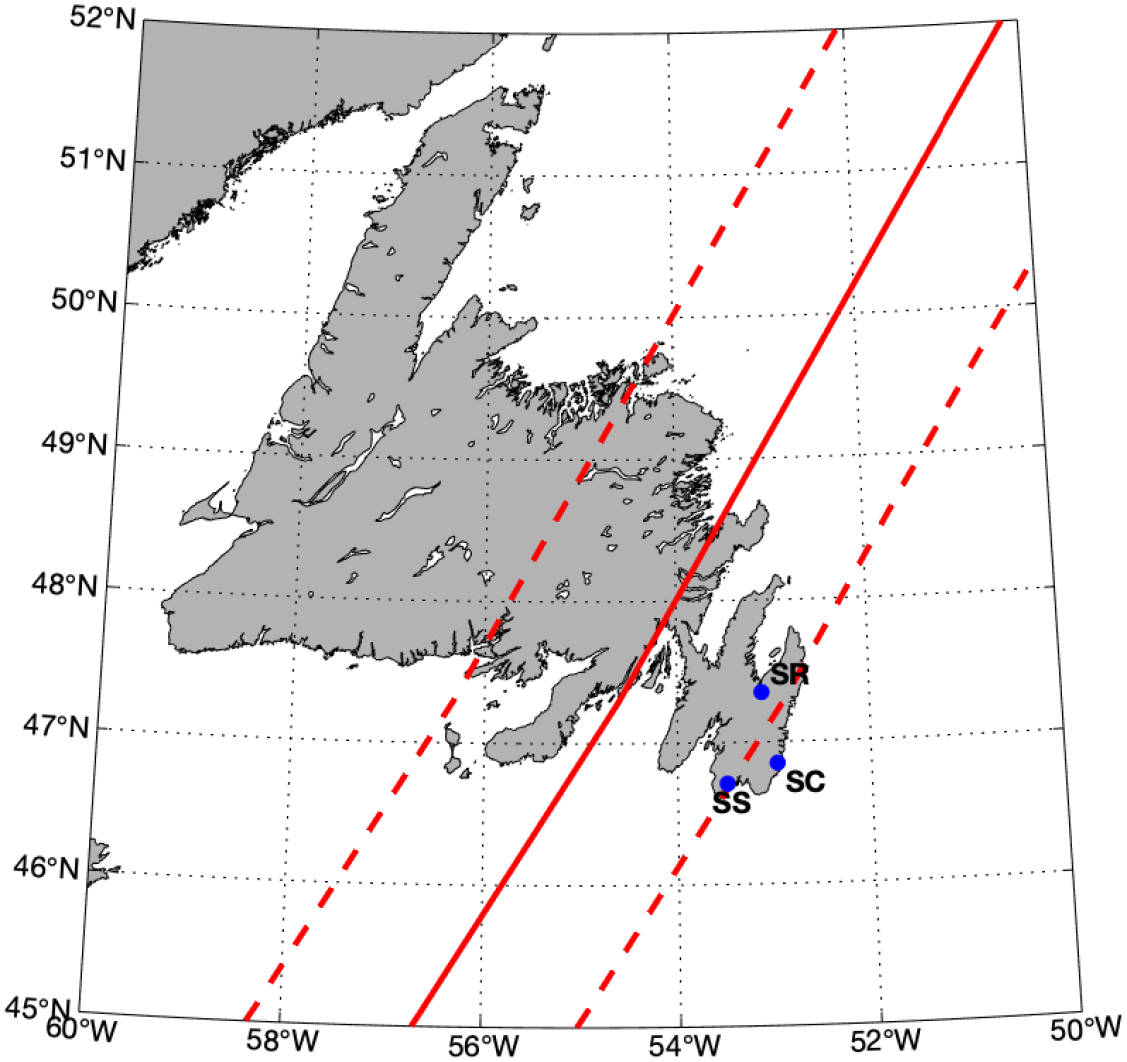
Path of Hurricane Larry (solid line) with extent of maximum winds (dashed lines) and sampling locations (blue dots). Hurricane path and wind extent data from the National Hurricane Center Atlantic HURDAT2 database [41]. Map created using M_Map package for MATLAB [42] using the NOAA GSHHG high-resolution coastline database [43].

Catchments in Newfoundland, Canada are dominated by peat barrens and boreal forest [39], both rich in organic carbon and iron. In September 2021, Hurricane Larry made landfall on the Avalon Peninsula of Newfoundland, Canada (Fig 1). This is the first hurricane within 11 years to reach the shores of Newfoundland, the previous one being Hurricane Igor in 2010. Hurricane Larry reached Newfoundland as a category 1 hurricane with a maximum sustained wind speed of 120 km/hr and gusts reaching a maximum of 180 km/hr. Rainfall was around 25–35 mm over a very short period of time causing localized flooding with significant storm surges with waves reaching 3.6 m [40].

While the island of Newfoundland experiences storm events with regular frequency, typically most of these events occur in the winter months, when productivity on land is limited and dissolved organic carbon (DOC) and iron concentrations are relatively low [44–48]. With the predicted increases in hurricane activity in the Atlantic Ocean, the island can expect to experience more of these events during the warm summer months, potentially leading to considerably increased carbon and iron export to the coastal zone that would not be experienced in a typical winter storm event.

Taking advantage of the well-predicted path of Hurricane Larry which made landfall in close proximity to Memorial University, we collected DOM samples from three rivers before and after landfall. We measured the change in concentration of DOC, iron, and colour to gauge the effects the hurricane has on the export of material from the island. Through the comparison of three rivers, one dominated by peatlands, one dominated by forest, and one with a mix of forest and peatlands we aim to identify how the different catchments react to the hurricane. The relationships between peatlands, forest and wetlands, and changes in DOM and iron export will be explored to see how landcover affects the catchment’s behaviour to extreme weather events. This is the first study measuring the effects of a hurricane on carbon cycling in Newfoundland and in the North American boreal environment. The limited number of sample sites and time points due to the opportunistic nature of this study prevents a more robust investigation into the mechanistic behaviours of the catchments. Rather, this serves as a preliminary exploration of how such environments will react to the threat of more extreme weather events brought on by climate change and warming oceans.

## 2. Methods

### 2.1. Sample Collection

Samples were collected from three river sites on the Avalon Peninsula of Newfoundland, Canada before (September 10^th^, 2021) and after the landfall of Hurricane Larry (September 11^th^, 2021). The three sites are equipped with water level and discharge gauges by Environment and Climate Change Canada (ECCC): Seal Cove Brook near Cappahayden, NL (SC, ECCC 02ZM009), St Shotts River near Trepassey, NL (SS, ECCC 02ZN002), and South River near Holyrood, NL (SR, ECCC 02ZM016). Water level and discharge data was extracted from the ECCC Real-time Hydrometric Data Website [49]. These three rivers were chosen out of fourteen gauged rivers on the Avalon Peninsula based on a number of factors. Of the fourteen rivers equipped with discharge and water level sensors, seven are in the heavily urbanized metro region making it unsuitable for such a study. Of the 7 remaining rivers, one is located downstream of a fish plant, while another is downstream of a mine, leaving 5 potential rivers to choose from. The three rivers chosen cover different landcover types of interest to the study, within safe driving distance given the dangers of sampling during an extreme weather event. SC has the longest historical record of discharge of the three rivers running continuously since 1979, SR started measuring discharge in 1983 with SS starting in 1985. The average discharge for the month of September spanning the historical range is calculated for the three rivers. No chemical parameters of the river such as temperature, pH, alkalinity, DOC, dFe, etc are collected at SR or SS. SC has a small chemical database spanning from 1986 to 1995 measuring lead and cadmium (Email communication with K. Brake Department of Environment and Climate Change, Government of Newfoundland and Labrador, November 2025).

Hurricane Larry made landfall shortly after midnight local time (23:00 Newfoundland Standard Time, NST) on September 11^th^, 2021. Samples were collected 12–18 hours after landfall as soon as it was determined that the road was passable to all three sites. There are no permits required for the collection of samples in any of the catchments as they all reside on public land. One litre of river water was collected at each site before and landfall, with the sample being stored in two 500 mL acid washed polycarbonate bottles to prevent iron and carbon contamination. The bottles were transported on ice back to the lab where they were filtered on the day of collection using ashed glass fibre filters (GF/F) filters (Whatman, nominal pore size 0.7 µm). Where appropriate, glassware was ashed (heated to 450°C for five hours) to burn off any residual carbon preventing carbon contamination. The filtered sample was then subdivided for dissolved iron (dFe), dissolved organic carbon (DOC), and a_350_ (a spectroscopic proxy for water colour and the presence of chromophoric DOM). Vials used for DOC, dFe, and a_350_ were stored in ashed vials capped with Teflon lined caps to further ensure sample integrity. Samples used for dFe and a_350_ were stored in amber coloured vials to reduce light degradation of iron or chromophoric DOM. Temperature and pH were measured in situ using a Thermo Scientific Orion Star A329 meter and pH probe.

### 2.2. Catchment characteristics

The catchments are dominated by boreal forest and peatlands, The three catchments occupy 2 distinct ecoregions, SR and SC are situated in the Maritime Barrens ecoregion and SS is situated in the Eastern Hyper-Oceanic Barrens ecoregion. The two ecoregions are distinguished by their proximity to the southernmost coast of the Avalon peninsula, while both regions are characterized by peat barrens and boreal tree species, the Eastern Hyper-Oceanic Barrens are exposed to strong winds, sea spray, and heavy fog most of the year [50]. Tree vegetation in the region is limited to sparse patches of tuckamore (or Krumholtz formations), which are low-lying shrub/thicket formations, as opposed to the typical though somewhat stunted boreal forest found in the Maritime Barrens. The breakdown of the landcover can be seen in Table 1.

**Table 1:** Landcover breakdown of the three catchments sampled.

Catchment Name	% forest	% peatland	% wetland	Catchment Area (km^2^)
SC	50.76	33.63	1.95	53.6
SR	33.90	44.84	1.28	17.3
SS	13.02	62.96	10.04	15.5

SC, Seal Cove Brook near Cappahayden; SR, South River near Holyrood; SS, St Shotts River near Trepassey.

Forest, peatlands, and wetlands are strongly associated with DOM and iron quality and quantity, and thus were chosen for statistical analysis for the changes in DOM before and after the Hurricane Larry [2,3,8,51–53]. Full landcover details can be found in the Government of Canada 2015 Land Cover of Canada report [39]. River discharge for the year for each catchment was used to calculate a relative Richard-Baker flashiness index (RB index) [54]. The RB index is a dimensionless indicator of how quickly a river returns to baseflow and can be used to compare catchment responses to storm events. This equation differs from the typical RB index equation being more suited for daily discharge comparisons, where q is the discharge of the river measured in m^3^/s, n is the total sample number and i represents the current sample in the calculation iteration [54].


$$\begin{array}{c} RB\;index=\frac{\sum_{i=\text{1}}^{n}\text{0}.\text{5}(\left|q_{i+\text{1}}-q_{i}\right|+|(q_{i}-q_{i-\text{1}}|)}{\sum_{i=\text{1}}^{n}q_{i}} \end{array}$$ (1)

### 2.3. Laboratory methods

#### 2.3.1. Dissolved organic carbon.

DOC was quantified as non-purgeable organic carbon on a Shimadzu TOC-L-CPH analyzer. The instrument was calibrated with a recrystalized acetanilide primary standard C_8_H_9_NO, Across Organics, 99 + % pure. The instrument measures samples using 3–5 injections ensuring a co-variance of 2% between injections, to ensure sample accuracy.

#### 2.3.2. Optical Properties.

DOM absorbance was measured on a Cary 300 spectrophotometer (Agilent), from 200–800 nm, with 1 nm resolution in a 5 cm quartz cuvette. Absorbance (A) was converted to decadal absorption (a) by *a* = A/0.05, where 0.05 is the pathlength of the cuvette in m. The absorption coefficient at 350 nm (*a*_*350*_) was used as a proxy for chromophoric DOM (CDOM). The conversion of absorbance to decadal absorption was calculated using MATLAB 2020A.

#### 2.3.3. Iron quantification.

Iron was quantified using the ferrozine method as outlined by Violler et al. [55] and Kritzberg et al. [3]. Briefly, 2.5 mL of sample is mixed with 515 µL of hydroxylamine hydrochloride solution and 250 µL ferrozine reagent and is then heated to just below boiling for 10 minutes. This step reduces all Fe to Fe (II) allowing complexation to the ferrozine reagent. Once the sample is removed from heat it is allowed to cool for 90 seconds before the pH is adjusted using 200 µL of basic ammonium acetate solution, and the resulting ferrozine-iron complex is measured by its absorbance at 562 nm. The method is calibrated using FeCl_3_. The FeCl_3_ calibration ranges from 0 to 5µmols/L using 6 points. The 6 points are 0,0.25µmol/L, 0.5µmol/L, 1µmol/L,2µmol/L and 5µmol/L. This method allows for a limit of detection of 0.0841µmol/L. To reduce the interference of DOM absorbance in Fe measurements, a reagent blank for each sample was also run. DOM of terrestrial origin can exhibit absorbance in the longer wavelength range and can add some interference to the iron measurements [56]. A reagent blank, i.e., running a sample with no reagents, can thus provide a correction factor for the iron measurements.

### 2.4. Statistical analysis

All statistics were carried out in MATLAB 2020A. After the hurricane, all parameters experienced a change: DOC, Fe, a_350_ increased while temperature, and pH experienced a decrease. To assess whether these changes were statistically significant one tailed paired t-tests (significance level of 0.05) were conducted. The t-test showed if the changes observed after the hurricane were statistically significant. One tailed t-tests were chosen based on the parameters having a clear direction of change, making it unnecessary to see if the variables changed in both directions which would be the case in a two-tail test. The paired t-test computes the difference in a measurement before and after treatment (the hurricane in this case) for each river separately before testing if the mean of the calculated differences of the full dataset varied from 0, to reject the null hypothesis of Ho = 0, where H_o_ is the mean of the calculated differences. The test computes a t-score which can be used to determine the p value and thus the significance of the change. In this way each river then serves as its own reference point, for an n = 3 dataset. The ∆ for the parameters were compared with landcover using Pearson’s correlations test. ∆ was calculated for each parameter and each individual river. The % coverage of the landcover types was individually compared to the ∆ of a parameter at a time, making an n = 3 dataset for each analysis. The low degrees of freedom for the Pearson’s correlation reduces statistical power the ρ are reported but the p-values are not as a result.

## 3. Results

### 3.1. Change in river state

**Fig 2:**
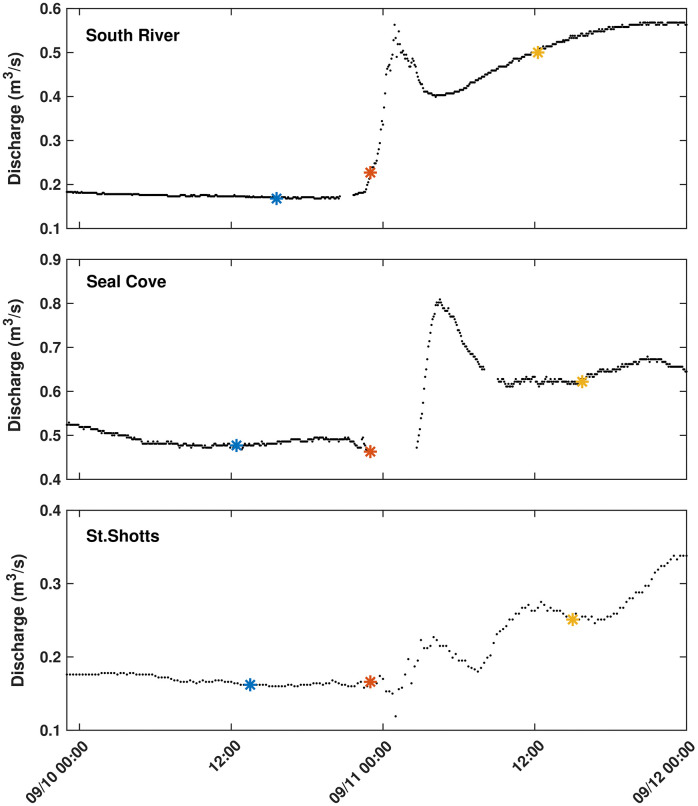
Hydrographs of the three catchments for the 24 hours preceding and following landfall of Hurricane Larry. The blue and yellow asterisks represent the time (NST) of sampling prior to and after landfall, respectively. The orange asterisk marks the time (NST) of landfall.

The hydrographs as seen in Fig 2, show that all rivers were in a period of stable baseflow for the 24 hours prior to the hurricane, experienced a peak in discharge within hours of landfall, and then remained in a high flow state for the 24 hours after landfall. In all three catchments, water level and discharge increased following the hurricane with discharge increasing by 235% in SR, followed by 70% for SC, and 40% for SS between the first sampling and the initial peak in discharge. In SC, the discharge stabilized after the initial discharge peak which occurred ~5.5 hours after landfall of the storm. In both SR and SS, discharge continued to increase after the initial peaks (2 and 5 hours after landfall, respectively) for the 24-hour period after landfall. While we were not able to sample at the time of the initial peaks, all rivers were sampled at higher discharges after the hurricane than before. The change in pH, temperature, discharge, and the RB index is shown in Table 2.

**Table 2:** Physical characteristics of the three rivers before and after Hurricane Larry, along with the historical discharge for the month of September. Exact times of sampling relative to landfall can be seen in Fig 2.

River	Time	Discharge (m^3^/s)	Temp(ºC)	pH	RB Index	Average historical discharge (m^3^/s)
**South River (SR)**	Before	0.168	20.5	7.16	0.3872	0.583
	After	0.500	19.4	6.82		
**Seal Cove (SC)**	Before	0.477	20.3	7.27	0.3722	2.101
	After	0.622	19.7	7.06		
**St Shotts (SS)**	Before	0.162	20.3	7.18	1.5719	0.505
	After	0.251	19.5	6.61		

**Fig 3:**
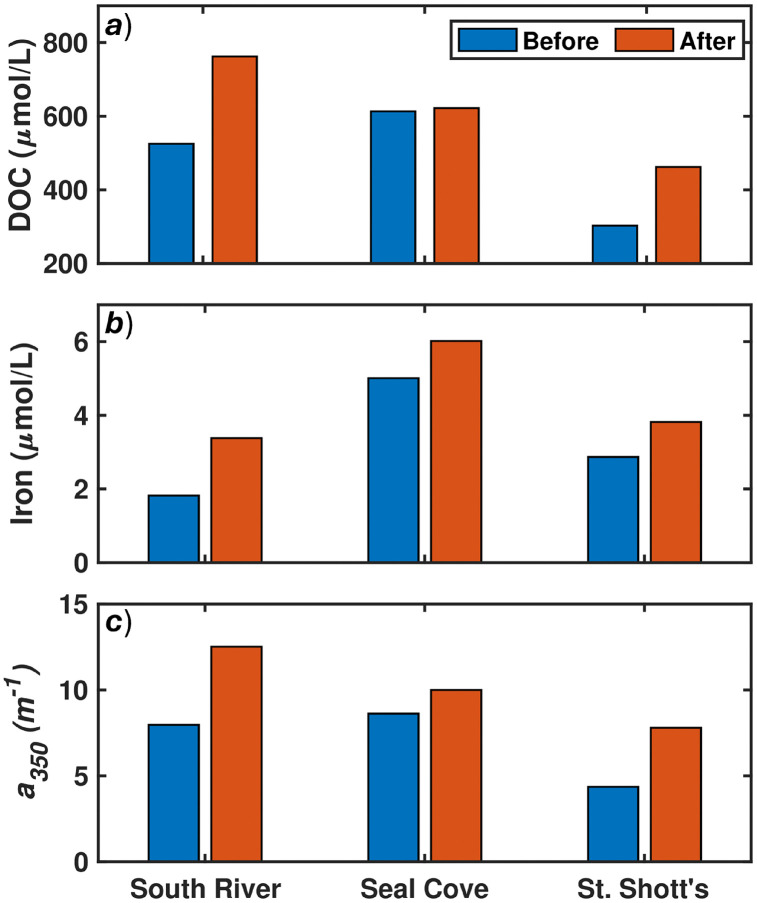
DOC concentrations, iron concentrations, and a_350_ before and after Hurricane Larry. a) dissolved organic carbon (µmol/L), b) iron (µmol/L), and c) a350 (m^-1^). Blue bar represents the before conditions with the red bar showing after Hurricane Larry.

Discharge is fairly low not exceeding 1m^3^/s at any point in the sample period (Table 2). The RB index can be used to compare the river’s response to storm events. SS is seen to be the flashiest returning to baseflow the quickest with SC taking the longest to return to baseflow. Both temperature and pH (Table 2) exhibited statistically significant decreases across all three catchments between sampling times (t-tests, p = 0.0145, p = 0.0356, respectively). Increases in DOC, iron concentrations and a_350_ were seen in all rivers as shown in Fig 3. Fe and a_350_ increases were found to be statistically significant (t-test, p = 0.0132, p = 0.0389, respectively) with DOC failing to achieve statistical relevance at the 0.05 significance interval (p = 0.0905). While the increase in DOC failed to meet the 0.05 significance interval it is important to highlight at a 0.1 significance interval it would have statistically significant differences. The DOC behaviours will still be discussed but they should not be seen as robust as the other parameters measured. DOC concentrations for SR and SS increased (+237 µmol/L and +159 µmol/L, respectively) with corresponding increases in *a*_*350*_ (+4.55 m^-1^ and +3.44 m^1^, respectively). Increase in iron was highest in SR (+1.57 µmol/L), and nearly the same in SC and SS (+1.01 µmol/L and +0.96 µmol/L, respectively).

### 3.2. Catchment characteristics and its effect on river conditions

Correlation analysis (Table 3) on the changes pre- and post- hurricane revealed that changes in DOC and *a*_*350*_ (i.e., ΔDOC and Δ*a*_*350*_) both had relatively strong negative correlations with the percent of peat in the catchment, as well as the total catchment area, and slightly less strong negative correlations to the percent forest cover. None of the correlations in the DOM analyses were statistically significant, due to the small dataset and low degrees of freedom. Neither ΔDOC nor Δ*a*_*350*_ correlated strongly with the amount of wetland in the catchment, with small negative relationships. In contrast, changes in iron (ΔFe) showed the opposite behaviour, with no relationship to forest or total catchment area, but a stronger negative relationship to the amount of wetland in the catchment and a weak negative correlation to peat. Change in discharge was negatively correlated to amount of wetland in the catchment (ρ = 0.998) and had no relationship to any other landcover variables.

**Table 3:** Pearson’s correlations (*ρ)* between changes in DOM properties and catchment land cover types.

	Peat	Forest	Wetland	Catchment Area	RB index
**Iron**	−0.567	−0.258	−0.960	−0.385	−0.569
**DOC**	−0.985	−0.869	−0.473	−0.928	0.187
***a***_**350**_	−0.983	−0.865	−0.481	−0.925	0.178

## 4. Discussion

Hurricane Larry made landfall in the late summer/early autumn of Newfoundland, a period where storms are not expected and where the productivity on land is peaking [44]. Following Hurricane Larry, all catchments experienced an increase in discharge, water level, and carbon and iron concentrations which is in line with current literature but the magnitude of change as well as the time it took to return to baselevel varied [12,14,18,27,57]. The difference in flashiness is expected based on the catchment size, land cover differences and catchment complexity [54]. SC, as the largest of the three catchments was the least flashy incorporating the increase in precipitation easily. SR had the highest increases in fluxes of material following landfall despite being the second largest catchment. The increase in flux highlights that in addition to catchment size, landcover plays a major role in catchment response. The differences in landcover appear to regulate the increases seen in the catchment behaviour. The relationships between different landcover types and parameters point to how the landcover controls the release of material following an extreme weather event. The limited sampling points reduces statistical power of tests investigating these relationships. The behaviours observed and discussed should be seen as a preliminary look into how the boreal region will react to such events. The various changes experienced by the rivers are discussed below.

### 4.1. River state

All three rivers experienced an increase in discharge following Hurricane Larry, but the discharge does not exceed historical averages for the month of September. All three rivers appear to have a much lower discharge compared to historical records prior to the arrival of the hurricane. When discharge is low the rivers are more likely to export DOM from the baseflow which is heavily dominated by groundwater inputs and deeper peat layers [14,58]. The decrease of pH is due to the increase of DOM entering the rivers. DOM from peatlands is known to be rich in carboxylic acids, an acidic compound, with more inputs from land the pH can be seen to decrease [59]. The decrease in temperature is due to the cloud cover and less thermal heating occurring following the storm.

### 4.2. Buffering of DOM and a_350_

Export of material following Hurricane Larry is buffered by the natural land cover present in all three catchments [60,61]. While the Pearson’s correlation failed to have high p values, the relatively high ρ values suggest there is a relationship to be explored with the collection of more data. The buffering capacity of natural landcover and storm events has been noted in prior literature [60]. The peat and forest appear to be the dominant source of DOM and colour to the waterways with iron being supplied by wetlands in the catchments. SS has more peat and wetland proportionally than the two other catchments. As the percentage of land cover increases there is a reduced Δ for the associated property. ΔDOC is buffered by an increase in the amount of peat present, a similar trend being seen for Δa350. The close relationship between DOC and a_350_ is to be expected as DOC provides highly coloured compounds around the 350nm range [62]. The buffering effect may be due to having a steady input of DOC from the peat layers. This buffering has been explored in past studies [58,63].The landscape of NL is dominated by peat and this can explain the small ∆s seen in response to extreme weather events such as Hurricane Larry. Typically, the deeper layers of peat provide DOM to the waterways through groundwater infiltration with the top layers only being accessed during storm events and overland flow [14,58]. With Newfoundland’s shallow soil and high-water table levels [64,65], the baseflow is expected to be more dominant and access a large portion of the soil column. Excess water will only act to speed up the export from the topsoil rather than access normally disconnected pools as in other catchments [12]. As these layers are capable of absorbing large quantities of water and are known to produce high levels of carbon, they are able to facilitate the extra water without significantly changing their export of DOC [58]. In systems such as these it is also common for a delayed peak to be observed where the water is absorbed and released later [57,66]. These delayed peaks could be the increase being seen in SR and SS following landfall.

### 4.3. Iron buffering

Iron is strongly correlated with the proportion of wetlands present. Iron is found to accumulate in such environments due to the saturated soils [27,51,53,67]. The saturated soils of wetlands are typically highly acidic and anoxic allowing for the accumulation of Fe (II) maintained in solution as opposed to precipitating out as iron hydroxides. The iron which can be built up in these reduced conditions can be quickly transported as the storm increases connectivity [27,51]. Like carbon, it is possible that with a high water table the pools of iron are already exporting material to the rivers through the river’s baseflow. Following the storm, the catchments with a high portion of wetland rather than access disconnected pools drastically increasing concentrations, the iron is more quickly mobilised leading to a smaller increase. In a catchment such as SS where the landscape is typically saturated a smaller increase may be seen as opposed to SR which comprises more forest areas. SS wetlands provide a consistent supply of iron to the river, and the increased precipitation facilitates an increase in iron mobilization only as opposed to SR. SR’s iron is mostly likely separated into different pools, mineral soils and wetlands, and the increased precipitation can increase hydraulic connectivity between these pools and thus lead to a greater increase in iron concentrations.

### 4.4. Eventual fate of the exports

Despite the increases being buffered by the presence of peat and wetlands the high concentrations of DOM and Fe present in these environments requires ongoing research to understand the controls on their fluxes. Studies have shown that the storm flux of DOM can either be mineralized or contribute to carbon storage based on the lability, and composition of the DOM and the microbial community present [9,10,13,20]. The fate of the DOM exported by Hurricane Larry is unknown.

The three rivers studied are all in catchments heavily dominated by boreal landscapes. The strong relationships between forest and peatlands and DOM seen in the t-test and Pearson’s correlations points to these landscapes acting as the dominant source of DOM to the catchment. While there are compositional differences between peatland-DOM and DOM from boreal forest there are key similarities which will affect the DOM fate in the coastal environment [28,58,68]. Both environments produce highly coloured DOM enriched in lignin and tannins which are microbially resistant to degradation [69]. The DOM exported by all three catchments should be high in these compounds and resistant to degradation. The formation of complexes with Fe in the boreal environment can aid in shielding DOM from microbial mineralization further ensuring the DOM exported should act as carbon sequestration [70,71] in the NL coastal environment. There are still gaps in understanding the behaviour of the boreal DOM through the estuary though with some DOM remaining in suspension while others will flocculate out in the estuary and contribute to carbon burial [72]. It is difficult to predict the eventual fate of this DOM as prior studies have focused on bays and large estuaries where different biogeochemical processes can occur with a longer residence time.

The study of carbon dynamics in estuaries in fact remains a key knowledge gap globally [7]. Small northern catchments such as these export high levels of DOM and Fe dismissing them can lead to underestimations in biogeochemical models [73,74]. The direct export of material from small catchments into the coast is understudied.

The lack of continuous monitoring during the hurricane and the inability to capture the initial peak discharge following landfall may have prevented us from capturing the exact dynamics present [19,47,75]. An increase in labile protein-like DOM has been observed in storm events being mobilized from the upper layers of peat [12,45]. This flush of labile protein has been seen in the first peak of outflow as it is quickly shunted to the coast in other studies. Due to sampling constraints, we were unable to capture the initial peak, missing key information following a storm event. The DOM exported is also expected to change on the falling limb of the hydrograph [57].Without continuous monitoring of these sites during storm events small but important changes in the dynamics of carbon and iron export may be missed.

## 5. Conclusions

Three catchments on the Avalon Peninsula of Newfoundland were sampled before and after Hurricane Larry made landfall. All three catchments are characterized by high levels of carbon and iron and all three increased their export following the hurricane. Our current results show that high intensity events such as Hurricane Larry will increase the export of material, but effects are buffered by saturated landcover. The landscapes prevalent in the boreal region impart resilience in carbon and iron exports, accommodating the increase in discharge and precipitation from extreme weather events. These results demonstrate the importance of natural landcover in regulating storm fluxes in the boreal landscape.

Due to the opportunistic nature of the study, sample sizes were small with only two time points at three rivers. Results from the study are not statistically robust in Pearson’s correlation but represent a preliminary look into the natural resilience of the boreal environment to extreme weather events. As climate change intensifies, it is likely that more hurricanes will affect the peat and boreal ecosystems found in the upper latitudes. Understanding how these environments will be affected by these intense storms will be pertinent as we consider the effects of global change on these ecosystems. The direct input of material into the coast should be studied further with higher-resolution data collection as the frequency of storm events reaching Newfoundland increases. High temporal resolution data that can be collected remotely can aid in capturing key moments in a catchment’s storm response, highlighting the processes occurring in these environments. Nevertheless this study represents the first study of high northern environments reacting to hurricane.
